# Blood pressure targets for prevention of cognitive decline in patients with diabetes and hypertension: Design of the Blood Pressure Control Target in Diabetes (BPROAD) Cognitive Study

**DOI:** 10.1111/1753-0407.13412

**Published:** 2023-09-22

**Authors:** The BPROAD Study Group, Guang Ning, Jiang He, Weiqing Wang, Yufang Bi, Dalong Zhu, Jiguang Wang, Shengdi Chen, Yu Xu, BPROAD coordinating team at Ruijin Hospital, Lawrence J. Appel, William C. Cushman, Vivian A. Fonseca, Jeff D. Williamson, David M. Reboussin, Yaling Han, Hongbing Shen, Minghui Zhao, Hui Wang

**Affiliations:** ^1^ Ruijin Hospital Shanghai; ^2^ Tulane University New Orleans; ^3^ Nanjing Drum Tower Hospital Nanjing; ^4^ Johns Hopkins University Baltimore USA; ^5^ University of Tennessee Memphis USA; ^6^ Tulane University New Orleans USA; ^7^ Wake Forest University Winston‐Salem USA; ^8^ General Hospital of Northern Theater Command Shenyang China; ^9^ Nanjing Medical University Nanjing China; ^10^ Peking University First Hospital Beijing China; ^11^ Shanghai Jiaotong University Shanghai China

**Keywords:** blood pressure, dementia, mild cognitive impairment, randomized controlled trial, type 2 diabetes, 血压, 痴呆症, 轻度认知障碍, 随机对照试验, 2型糖尿病

## Abstract

**Background:**

Both hypertension and diabetes are risk factors of dementia. Proper management of blood pressure (BP) and blood glucose is critical in delaying cognitive decline in the elderly. However, little is known regarding the optimal BP target in type 2 diabetes (T2DM) for the management of cognitive decline.

**Methods:**

The Blood Pressure Control Target in Diabetes (BPROAD) study is a nationwide, multicenter, randomized controlled trial that will enroll 12 702 T2DM patients with elevated systolic BP and increased cardiovascular risk from approximately 150 study centers across mainland China to undergo randomly antihypertensive treatment achieving systolic BP <120 mm Hg or systolic BP <140 mm Hg for up to 5 years. All BPROAD participants will take part in the BPROAD Cognitive Study for the assessment of cognitive function at baseline and annual visits by blinded outcome assessors to determine whether intensive BP treatment reduces risk of dementia and mild cognitive impairment (MCI) compared with standard BP treatment in patients with T2DM. In addition, approximately 1000 BPROAD participants will be enrolled in the magnetic resonance imaging (MRI) substudy to receive brain MRI at baseline and at closeout. The primary outcome of BPROAD Cognitive Study is a composite of all‐cause dementia and MCI.

**Conclusions:**

The BPROAD Cognitive Study will provide crucial clinical trial data on the possible benefit of an intensive systolic BP lowering strategy in reducing dementia and MCI in patients with T2DM.

## Graphical abstract

Highlights

- The optimal blood pressure (BP) target in patients with type 2 diabetes mellitus (T2DM) for the management of cognitive decline remains unknown.
- The Blood Pressure Control Target in Diabetes (BPROAD) Cognitive Study was designed to determine whether intensive BP treatment (systolic BP <120 mm Hg) reduces risk of dementia and mild cognitive impairment compared with standard BP treatment (systolic BP <140 mm Hg) in patients with T2DM.
- Findings from the study will have essential impacts on public health and clinical practice of BP management in patients with T2DM.

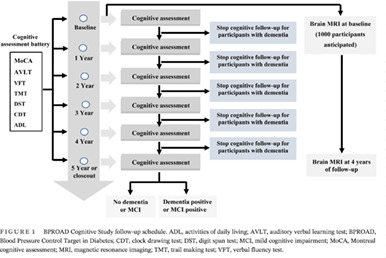

## 摘要

背景:高血压和糖尿病都是痴呆症的风险因素。适当地管理血压(BP)和血糖是延缓老年人认知下降的关键。然而，对于2型糖尿病(T2DM)认知减退管理的最佳BP目标值知之甚少。

方法:BPROAD研究是一项全国性多中心随机对照试验，将招募12,702名T2DM患者。这些患者来自中国大陆约150个研究中心，患者的收缩压升高，心血管风险增加。所有BPROAD参与者将参加BPROAD认知研究，以评估基线和年度访问时的认知功能，由盲态的结果评估员确定与标准BP治疗相比，强化BP治疗是否可降低T2DM患者的痴呆和轻度认知障碍(MCI)风险。此外，约1,000名BPROAD参与者将参加磁共振成像(MRI)亚组研究，在基线和4年随访中接受脑部MRI检查。BPROAD认知研究的主要结果是所有原因的痴呆症和MCI的复合指标。

结论:BPROAD认知研究将为减少T2DM患者的痴呆症和MCI提供关键的临床试验数据。

## 1 INTRODUCTION

Type 2 diabetes mellitus (T2DM) has become a critical health concern worldwide.^1^ A causative association between diabetes and cognitive impairment has been suggested.^2^ T2DM is associated with increased blood viscosity, impairment of vascular endothelial function, and abundance of inflammatory mediators, thereby leading to vascular stenosis, microcirculatory hemodynamic disorders and neurodegeneration. T2DM is associated with a 148% increased risk of vascular dementia and a 46% increased risk of Alzheimer's disease.^3^ Hypertension is also one of the strongest risk factors for dementia.^4^ Long‐term hypertension induces vascular atherosclerosis, arterial aneurysm, inflammation, and changes in cerebral hemodynamics, which lead to ischemia and hypoxia in the brain.^5^ This process ultimately results in neuron loss and development of vascular dementia. Cognitive function decreased more rapidly in elderly patients having both T2DM and hypertension.^6^

Few studies have examined the impact of blood pressure (BP) control on cognitive function among patients with T2DM. The Memory in Diabetes (MIND) substudy of the Action to Control Cardiovascular Risk in Diabetes (ACCORD) trial found that there was no significant difference in cognitive outcomes between intensive BP treatment targeting systolic BP < 120 mm Hg and standard BP treatment targeting systolic BP < 140 mm Hg in patients with T2DM and hypertension. However, the total brain volume evaluated by the magnetic resonance imaging (MRI) reduced more in the intensive group.^7^ No statistically significant difference was found for probable dementia between intensive and standard BP lowering groups in patients without diabetes in the Systolic Blood Pressure Intervention Trial Memory and Cognition in Decreased Hypertension (SPRINT MIND). However, the study was terminated early due to significant benefits of lowering cardiovascular risk with intensive BP treatment. Therefore, fewer than expected cases of dementia were observed and the study was underpowered for this end point. In addition, risks of secondary cognitive outcomes including mild cognitive impairment (MCI) and the combined MCI or dementia were reduced significantly in the intensive BP treatment group.^8^ In the imaging substudy of SPRINT MIND, intensive BP control was significantly associated with a smaller increase in cerebral white matter lesion volume but a greater decrease in total brain volume compared with standard BP control.^9^

Both ACCORD MIND and SPRINT MIND studies have limitations. The ACCORD MIND study was not designed to assess adjudicated MCI or dementia, and SPRINT MIND excluded patients with T2DM. Therefore, whether intensive BP control benefits cognitive function in T2DM remains to be answered. The Blood Pressure Control Target in Diabetes (BPROAD) Cognitive Study was designed to test whether lowering systolic BP to <120 mm Hg reduces a combined risk of dementia and MCI compared with lowering systolic BP to <140 mm Hg in patients with T2DM.

## 2 OBJECTIVES

The BPROAD study is a nationwide, multicenter, outcome assessor blinded, randomized controlled trial (ClinicalTrials.gov Identifier: NCT03808311) that will enroll 12 702 T2DM patients with elevated systolic BP and increased cardiovascular risk from approximately 150 study centers across mainland China. The overall goal of BPROAD is to determine whether randomization to an antihypertensive treatment strategy achieving systolic BP <120 mm Hg is more effective than a treatment strategy achieving systolic BP <140 mm Hg in reducing the risk of major cardiovascular events (a composite of nonfatal stroke, nonfatal myocardial infarction, hospitalized or treated heart failure, and cardiovascular death) among patients with hypertension and T2DM over a follow‐up period of up to 5 years.

The cognitive component of the BPROAD study, the BPROAD Cognitive Study, was designed to determine whether intensive treatment to systolic BP <120 mm Hg is more effective than standard treatment to systolic BP <140 mm Hg in reducing the risk of a composite outcome of all‐cause dementia and MCI among patients with T2DM. In addition, other objectives are to compare between intensive and standard BP treatment groups:

- the occurrence of all‐cause dementia;
- the occurrence of MCI;
- changes in global and specific cognitive domains including learning and memory, complex attention, executive functioning, language, and visuospatial skills; and
- changes in total brain volume, incidence of cerebral small vessel disease, etc. assessed by brain MRI.

## 3 STUDY POPULATION

BPROAD participants will be recruited from secondary or tertiary hospitals across mainland China. The inclusion and exclusion criteria for BPROAD participants are described in detail in the design paper of the BPROAD study. We will enroll T2DM patients aged ≥50 years with elevated systolic BP and increased cardiovascular risk. All BPROAD participants will receive a cognitive assessment at the baseline visit and will be reassessed at the annual follow‐up visits and the close‐out visit. In addition, 5–10 BPROAD study centers will be selected for the MRI substudy based on MRI scanner types and willingness to participate. BPROAD participants at these centers will undergo brain MRI at baseline and at closeout. Participants without written informed consent for MRI examination and participants with conditions judged by a radiologist not appropriate for brain MRI assessment will be excluded from the MRI substudy.

The study design and study documents were approved prior to use by the Institutional Review Board (IRB) of Ruijin Hospital, as well as by the IRBs of participating study centers. Written informed consent is obtained from all study participants prior to participation.

## 4 FOLLOW‐UP VISITS

**FIGURE 1:**
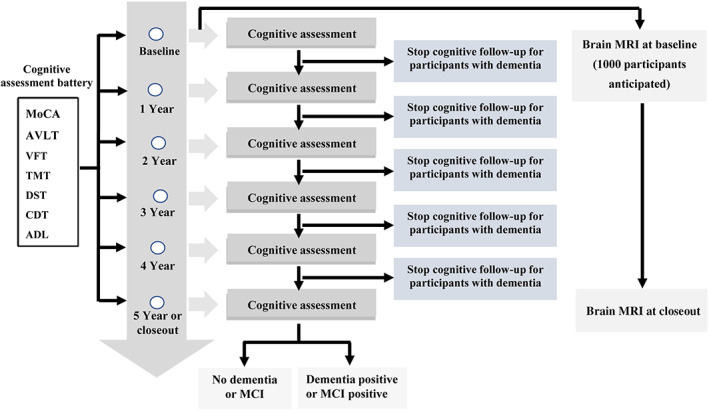
BPROAD Cognitive Study follow‐up schedule. ADL, activities of daily living; AVLT, auditory verbal learning test; BPROAD, Blood Pressure Control Target in Diabetes; CDT, clock drawing test; DST, digit span test; MCI, mild cognitive impairment; MoCA, Montreal cognitive assessment; MRI, magnetic resonance imaging; TMT, trail making test; VFT, verbal fluency test.

The follow‐up visit schedule for data collection does not differ by treatment group assignment. Cognitive assessments will be administered to all BPROAD participants at the baseline visit, at annual follow‐up visits, and at the close‐out visit (Figure 1). Participants in the MRI substudy will receive brain MRI examination at the baseline visit and at closeout. Study physicians performing cognitive assessments at each study center are unaware of participants' randomization assignments.

## 5 COGNITIVE ASSESSMENT

The test battery, which requires approximately 45 minutes to complete, included the Montreal Cognitive Assessment (MoCA) test for global cognitive function and other tests for domain cognitive function. Five cognitive domains will be evaluated including learning and memory, complex attention, language ability, executive functioning, and visuospatial skills. All the cognitive tests are in Chinese version and have been validated in the Chinese population. All the tests were administered and scored according to standard procedures.

- MoCA

In the BPROAD Cognitive Study, we use the MoCA to assess global cognitive function. It assesses different cognitive domains including attention and concentration, executive functions, naming, memory, language, visuoconstructional skills, conceptual thinking, calculations, and orientation. We will use the Mandarin version of MoCA (v7.1). According to a validation study, the sensitivity of the MoCA for detecting MCI was 80.5%, and the specificity for identifying normal cognitive status was 82.5%, respectively.^10^

- Auditory verbal learning test (AVLT)

We use the AVLT‐Huashan version to assess learning and memory. The AVLT‐Huashan version was compiled based on the California Verbal Learning Test and the Chinese University of Hong Kong Verbal Learning Test in 1998. The AVLT assesses basic processes of brain encoding, storing and retrieving of memories. There are three immediate recall trials, followed by the short‐term delayed free recall, long‐term delayed free recall, long‐term delayed cued recall, and Yes/No recognition trials.^11^

- Verbal fluency test (VFT)

We use the VFT to assess language ability and it is also a measure of retrieval from semantic memory. The VFT originated from the Controlled Verbal Fluency Test (CVFT).^12^ In VFT, participants are given 1 min to produce as many unique words as possible based on a given set of rules (eg, generate as many names of animals as possible). The participant's score in each task is converted based on the number of unique correct words.

- Trail making test (TMT)

We use the modified TMT to assess executive functioning. The test consists of two parts: the TMT‐A (Shape Trail Test) and TMT‐B (Color Trail Test).^13^ In TMT‐A, participants are instructed to connect a set of 25 numbers as quickly as possible while still maintaining accuracy. In TMT‐B, the numbers are colored with red or yellow. The test can provide information on visual search speed, scanning, speed of processing, mental flexibility as well as executive functioning.

- Digit span test (DST)

We use the DST, which is a subtest from the Wechsler Memory Scale to assess complex attention. After being read a series of numbers, participants are told to repeat the numbers in forward or backward order. The forward DST assesses both attention and short‐term memory, and the backward DST also measures working memory.

- Clock drawing test (CDT)

We use the CDT to assess visuospatial skills. Participants are required to draw a clock with all the numbers and pointers making a particular time. The test also evaluates executive functions by integrating spatial, digital and temporal capacities.

- Activities of daily living (ADL)

The decline in activities of daily living is a core symptom of dementia. In the BPROAD Cognitive Study, we use the ADL‐Zhang Mingyuan Revised Version to assess a participant's functional status.^14^ The ADL contains 20 items, including physical self‐maintenance and instrumental activities of daily living. Unlike other tests, the ADL is completed by a family member who is familiar with the patient's activities of daily living.

## 6 BRAIN MRI

In order to participate in the MRI substudy, study centers should have a 3.0 T MRI scanner with following pulse sequences:

- Sequence 1 – 3‐plane gradient echo localizer for positioning,
- Sequence 2 – Sagittal 3D T1‐weighted sequence for entire brain coverage,
- Sequence 3 – Sagittal 3D FLAIR images from matching slice positions in Sequence 2,
- Sequence 4 – Axial BOLD (blood oxygen level dependent) functional MRI sequence (resting),
- Sequence 5 – Axial ASL (arterial spin labeling) perfusion sequence,
- Sequence 6 – SWI (susceptibility weighted imaging) sequence, and
- Sequence 7 – Time of flight magnetic resonance angiography.

Each MRI study center has a BPROAD MRI technologist to ensure protocol compliance, resolve technical issues, and facilitate communication.

## 7 OUTCOMES

Pictures of completed cognitive tests are uploaded to the electronic data capture system. Each test is rated independently by two staff members at the BPROAD Coordinating Center at Ruijin Hospital who are blinded to participants' treatment assignment according to a standard procedure. A cognitive outcome adjudication committee of neurologists with expertise in cognitive neurology adjudicates all possible dementia and MCI cases. Two committee members independently review each participant's test performance and made adjudications. Disagreements are discussed by all committee members in adjudication meetings. Adjudicators are blinded to treatment assignment.

### 7.1 Primary outcome

The primary outcome of the BPROAD Cognitive Study is a composite of all‐cause dementia and MCI. Dementia is defined according to the Diagnostic and Statistical Manual of Mental Disorders‐Fourth Edition (DSM‐IV)^15^:

- Significant decline in memory and at least one additional cognitive domain; and
- Significant functional impairment due to cognitive problems; and
- Cognitive deficits are not due to obvious reversible causes.

No attempt to classify dementia subtypes will be made.

MCI is defined according to the 2011 Recommendations from the National Institute on Aging‐Alzheimer's Association workgroups on diagnostic guidelines for Alzheimer's disease^16^:

- Concern regarding a change in cognition; and
- Impairment in one or more cognitive domains; and
- Preservation of independence in functional abilities; and
- Not demented.

Two occurrences of an adjudicated classification of MCI will be required for the MCI diagnosis. The second adjudication of MCI need not to be consecutive. Similarly, MCI then a later dementia adjudication is also acceptable for the MCI diagnosis.

### 7.2 Secondary outcomes

- All‐cause dementia
- MCI
- Changes in global cognitive function and in five specific cognitive domains
- Changes of brain structure

Changes in brain MRI characteristics will be compared between treatment groups in the MRI substudy.

- Changes in total brain volume
- Number of high signal lesions of white matter in brain
- Occurrence of cerebral small vessel disease

## 8 STATISTICAL POWER

All BPROAD participants will be included in the Cognitive Study and we will recruit a total of 12 702 participants. Because of the greater lag in the effects of BP reduction on cognitive effects than on CVD effects, the end of the trial for the cognitive component will likely occur after the end of the trial for the CVD outcome. The SPRINT MIND study observed a 2.4% per year event rate of a combined dementia and MCI in the standard treatment group. Because we may have a study population younger than the SPRINT MIND population, we assume a range between 1.8% to 2.4% per year event rate of the primary cognitive outcome and calculate the anticipated total event number and statistical power at different annual event rates and total study lengths shown in Table 1. The BPROAD Cognitive Study will continue until 1374 cases of dementia or MCI (85% power) are observed.

**TABLE 1:** Anticipated number (statistical power) of the primary cognitive outcome events.

Total study length	Event rate (% per year)						
	1.8	1.9	2.0	2.1	2.2	2.3	2.4
5 years	785 (62%)	827 (65%)	869 (67%)	911 (69%)	952 (71%)	994 (73%)	1035 (74%)
6 years	964 (71%)	1015 (73%)	1066 (76%)	1117 (77%)	1168 (79%)	1218 (81%)	1268 (82%)
7 years	1137 (78%)	1197 (80%)	1256 (82%)	1315 (84%)	1374 (85%)	1433 (87%)	1491 (88%)

*Note*: 15% effect size for the intervention (hazard ratio of 0.85); 2‐year recruitment with a uniform accrual rate; annual proportion lost to follow‐up = 2%; two‐sided significance level = 0.05; and an overall sample size = 12 702.

## 9 STATISTICAL ANALYSIS

The primary analysis will be based on participants' randomization assignment regardless of their achieved BP levels (intention‐to‐treat analysis). The primary endpoint for BPROAD Cognitive Study is time to first identification of all‐cause dementia or MCI. Cumulative event rates of all‐cause dementia and MCI will be calculated using the Kaplan–Meier method and differences between the two treatment groups will be tested using the log‐rank test. The Cox proportional‐hazards model will be used to compare the time from randomization to the first identification of dementia or MCI between the two treatment groups. The model will include an indicator for intervention as its sole predictor variable. Study center at randomization will be a stratifying factor. Effect estimates (hazard ratios) with 95% confidence intervals will be reported. The changes in global and domain‐specific cognitive performance will be calculated as *Z* scores standardized to the baseline mean and SD at each time point. We will use mixed‐effects regression models with maximum likelihood estimation to assess between‐group differences in changes from baseline to the last visit.

The following subgroups are prespecified in the BPROAD Cognitive Study.

- Age at baseline (<75 vs ≥75 years)
- Men vs women
- Systolic BP levels at baseline (tertiles)
- Previous cardiovascular disease (CVD)
- Previous chronic kidney disease
- Glycated hemoglobin (HbA1c) levels at baseline (tertiles)
- Standing systolic BP (tertiles)
- Apolipoprotein E (APOE) ε4 allele (0, 1, 2)

## 10 QUALITY CONTROL

The BPROAD Manual of Procedures includes detailed descriptions of cognitive test procedures and is used for training purposes and as a reference for study investigators and staff. Physicians who are examiners of cognitive function receive a half‐day, centrally administered, face‐to‐face training including detailed instructions on the conduct of each cognitive test, hands‐on training of cognitive assessments, and finally a quiz. A training video is also provided to all study centers. In addition, the Coordinating Center will organize yearly refresher training sessions on cognitive assessments.

All cognitive tests will be conducted at BPROAD study centers according to standard procedures as outlined in test manuals. Quality control will be made at the Coordinating Center by examination of completed cognitive tests in the electronic data capture system. Data queries regarding proper conduct of cognitive tests and abnormal or missing values will be sent to study centers for a timely correction or explanation. The Coordinating Center will send monthly report to each study center summarizing problems and providing Q&As.

For the MRI substudy, radiologists at participating centers receive face‐to‐face training on standard procedures of brain MRI scanning. A pilot examination of a healthy volunteer is conducted and MRI images are reviewed by a group of experienced radiologists at the Coordinating Center, before enrolling BPROAD participants for brain MRI examination. During the study, MRI images of BPROAD participants will be stored on hard drives and sent back to the Coordinating Center on a regular basis.

## 11 CONCLUSIONS

Both hypertension and diabetes are risk factors of dementia. Patients with both diseases are at higher risks of cognitive decline. Findings from previous studies such as the ACCORD MIND and the SPRINT MIND have provided first evidence regarding intensive vs standard BP reduction on cognitive outcomes. However, the question whether intensive BP treatment lowers the risk of cognitive decline remains largely unanswered. In this regard, the BPROAD Cognitive Study will have important public health and clinical implications. It will provide crucial clinical trial data on the possible benefit of an intensive systolic BP lowering strategy in reducing dementia and MCI in patients with T2DM.

## CONFLICT OF INTEREST STATEMENT

None.
